# A systematic review and meta-analysis of the timing of rehabilitation training intervention for recipients of extracorporeal membrane oxygenators

**DOI:** 10.3389/fmed.2026.1919668

**Published:** 2026-10-05

**Authors:** Zhen Cheng, Liping Du, Shuangqin Zheng, Xiaoheng Zheng, Lingling Cheng, Mengfei Zhang, Chenmo Shi, Tianyu Zhang, Wei Zheng, Aili Shi

**Affiliations:** 1 Emergency and Intensive Care Unit Center, Department of Emergency Medicine, Zhejiang Provincial People's Hospital (Affiliated People's Hospital, Hangzhou Medical College), Hangzhou, Zhejiang, China; 2 Emergency and Intensive Care Unit Center, Intensive Care Unit, Zhejiang Provincial People's Hospital (Affiliated People's Hospital, Hangzhou Medical College), Hangzhou, Zhejiang, China; 3 School of Public Health and Nursing, Hangzhou Normal University, Hangzhou, China; 4 Tongde Hospital of Zhejiang Province Affiliated to Zhejiang Chinese Medical University College of Integrated Traditional Chinese and Western Medicine Clinical Medicine), Hangzhou, China

**Keywords:** critical care, early rehabilitation, extracorporeal membrane oxygenation, meta-analysis, rehabilitation

## Abstract

**Objective:**

To systematically evaluate the effects of early versus delayed rehabilitation training interventions (RTI) on clinical outcomes in patients receiving extracorporeal membrane oxygenation (ECMO).

**Methods:**

We conducted a systematic review and meta-analysis of randomized controlled trials (RCTs), non-randomized controlled studies, and observational cohort studies comparing early and delayed RTI in patients receiving ECMO. We searched databases including Embase, PubMed, Web of Science, ScienceDirect, China National Knowledge Infrastructure, China Science and Technology Journal Database, and China Biology Medicine disc from inception to 1 April 2025. Study selection, data extraction, and quality assessment were performed independently by two reviewers. Outcomes included complications, intensive care unit (ICU) and hospital length of stay (LOS), duration of mechanical ventilation, ECMO duration, and in-hospital mortality.

**Results:**

Twelve studies (five RCTs, four non-RCTs, and three cohort studies) were included. Compared with delayed RTI, early RTI significantly reduced the risk of complications [risk ratio (RR) = 0.43, 95% CI: 0.26–0.69, *P* < 0.001], shortened ICU LOS [mean difference (MD) = −1.12 days, 95% CI: −1.30 to −0.94, *P* < 0.001], and reduced hospital LOS (MD = −4.69 days, 95% CI: −8.11 to −1.27, *P* = 0.007). No significant differences were found in mechanical ventilation duration (MD = 0.14 days, *P* = 0.914), ECMO duration (MD = −1.77 days, *P* = 0.136), or in-hospital mortality (RR = 0.72, *P* = 0.097). Sensitivity analysis excluding one study demonstrated a greater reduction in hospital LOS significant (MD = −5.82 days, 95% CI: −9.34 to −2.30, *P* = 0.001), while other outcomes remained unchanged.

**Conclusions:**

Early RTI is associated with reduced complications and improved recovery outcomes (ICU/hospital stay) in ECMO patients compared with delayed RTI. However, due to the limited number and moderate quality of included studies, these findings should be interpreted with caution. High-quality RCTs are needed to confirm these results.

**Systematic Review Registration:**

https://www.crd.york.ac.uk/prospero/display_record.php?ID=CRD420251025533, PROSPERO CRD420251025533.

## Introduction

Extracorporeal membrane oxygenation (ECMO) provides temporary cardiopulmonary support for patients with severe cardiac or respiratory failure and during extracorporeal cardiopulmonary resuscitation, among other clinical scenarios (1), temporarily substituting for the function of the heart and lungs (2). There are two main types: veno-arterial (VA)-ECMO and veno-venous (VV)-ECMO. VA-ECMO draws blood from the venous system, passes it through an oxygenator, and returns it to the arterial system, providing both circulatory and respiratory support; VV-ECMO, on the other hand, draws blood from the venous system and reinfuses it back into the venous system, providing only respiratory support (3). During the global spread of COVID-19, use of ECMO was recommended as a primary treatment for patients suffering from refractory acute respiratory distress syndrome (ARDS) (4).

Due to prolonged bed rest and their critical condition, patients receiving ECMO are at high risk of severe muscle weakness and functional decline, resulting in prolonged mechanical ventilation duration, ICU length of stay, hospitalization duration, and long-term physical disability (5).

RTIs have been proposed to mitigate these effects in critically ill patients (6). Early RTI refers to interventions initiated immediately or within 24–48 h after ECMO initiation and continued for up to 4 weeks, including both passive and active exercise (7, 8). Ferreira et al. (9) analyzed 317 patients receiving ECMO who underwent early RTI such as respiratory therapy, early progressive activity, and muscle electrical stimulation, and reported that early RTI is relatively effective and safe for ECMO patients. However, a randomized controlled trial (RCT) by Hayes et al. (10) showed that early RTI during ECMO had no significant effect on patient physiological parameters, nor did it significantly shorten patients’ ICU and hospital length of stay. A systematic review by Chatziefstratiou et al. (11) included eight relevant studies and suggested that early RTI was associated with reduced hospitalization duration, decreased duration of mechanical ventilation, and lower vasopressor doses. However, this review lacks quantification and meta-analysis of data. To resolve this controversy, we conducted a systematic review and meta-analysis of controlled studies, to evaluate the timing and effectiveness of early RTI in patients receiving ECMO.

## Materials and methods

This meta-analysis was conducted and reported in accordance with the PRISMA statement for randomized controlled trials and the MOOSE guidelines for observational studies. The study protocol was prospectively registered in PROSPERO (registration number CRD420251025533), and the publicly accessible record includes comprehensive details on the search strategy, study objectives, eligibility criteria, data extraction, and quality assessment of the included studies. No amendments were made to the registered protocol.

### Databases and search strategy

We searched Embase, PubMed, Web of Science, ScienceDirect, China National Knowledge Infrastructure, China Science and Technology Journal Database, China Biology Medicine, and Google Scholar from database inception to 10 April 2025. The search keywords included “Extracorporeal Membrane Oxygenation,” “ECMO,” “VV-ECMO,” “VA-ECMO,” “Early rehabilitation training intervention,” “Early Physical Therapy,” “Early rehabilitation,” “Early mobilization,” “early physical rehabilitation,” and “early initiation of physical therapy.” The full search strategies for PubMed and Embase are provided in the Supplementary Material.

The search formula for advanced search in PubMed was:

(“Extracorporeal Membrane Oxygenation”[Mesh] OR “ECMO”[tiab] OR “Extracorporeal Life Support”[tiab] OR “VV-ECMO”[tiab] OR “VA-ECMO”[tiab] OR “Extracorporeal Membrane Oxygenations”[tiab] AND (“Early rehabilitation training intervention”[tiab] OR “Early Physical Therapy”[tiab] OR “Early Ambulation”[tiab] OR “Endurance Training”[tiab] OR “Early Physical Rehabilitation”[tiab] OR “Early mobilization”[tiab])

The search formula for advanced search in Embase was:

(‘Extracorporeal Membrane Oxygenation’/exp OR ‘ECMO*’:ti,ab,kw) AND (‘Early rehabilitation training intervention’:ti,ab,kw OR ‘Early Physical Therapy’:ti,ab,kw OR ‘Early Ambulation’:ti,ab,kw OR ‘Resistance Training’:ti,ab,kw OR ‘Exercise Therapy’:ti,ab,kw OR ‘Endurance Training’:ti,ab,kw OR ‘Physical Rehabilitation’:ti,ab,kw OR ‘Early Physical Rehabilitation’:ti,ab,kw)

### Eligibility criteria

The following criteria were used for inclusion: the design type of the trial is a randomized controlled study trial, non-randomized controlled study, or observational cohort study; early RTI was defined according to individual study protocols as rehabilitation initiated within 7 days after ECMO initiation (with specific start times ranging from within 24 h to 1–3 days across studies), or immediately after ECMO decannulation in selected protocols; the recruited patients are ECMO users, including VV-ECMO and VA–ECMO; the primary diseases of the patients included severe cardiopulmonary conditions, such as severe COVID-19 infection, congestive heart failure, chronic lung disease, and explosive myocarditis; the intervention group received early RTI, while the control group received delayed RTI (Intervention was initiated 7 days later); and at least one predefined outcome was reported, including ECMO duration, mechanical ventilation duration, length of hospital stay, ICU length of stay, incidence of complications, or mortality rate.

The exclusion criteria are as follows: non-ECMO patients; case reports, reviews, and commentaries; unfinished report.

### Study screening

After retrieving the initial search records from the databases and removing duplicate publications, two reviewers independently screened the titles and abstracts, and full texts of the articles based on the inclusion and exclusion criteria. Any discrepancies were resolved by consulting with a third author.

### Quality assessment

Risk of bias was assessed using the Cochrane Risk of Bias Tool (RoB 2.0) (12) for RCTs and the ROBINS-I tool for non-randomized studies. Each of which was evaluated as “low,” “some concern of risk,” and “high.”

### Data extraction

Two researchers conducted data extraction of existing literature using a structured form, covering variables such as author, publication year, country, trial duration, trial registration identifier, sample size, gender, age, follow-up duration, and outcome measures.

### Outcomes and effect size

The primary outcome measures were ultimately determined as the hospital length of stay (LOS), ICU LOS, duration of ECMO, mechanical ventilation duration, incidence of complications, and in-hospital mortality. For hospital LOS, ICU LOS, ECMO duration, and mechanical ventilation time, the mean difference (MD) was used as the effect size for comparisons between early RTI and delayed RTI, while for complication incidence and in-hospital mortality, the risk ratio (RR) was adopted as the effect size.

### Statistical analysis

Meta-analysis was performed using RevMan 5.4. Continuous outcomes were pooled using MDs, and dichotomous outcomes using RRs, with 95% CI. Heterogeneity was assessed using *I*^2^ and Cochrane Q tests. A random-effects model was used if *I*^2^ > 50%; otherwise, a fixed-effects model was applied. Sensitivity analysis and publication bias assessment were conducted. Sensitivity analysis was conducted using the leave-one-out method. Egger’s test was used to assess publication bias and the results were visually presented in a funnel plot. A *P*-value less than 0.05 was considered statistically significant.

## Results

### Study selection

A total of 757 articles were retrieved from the online databases, and 133 articles were obtained from searches conducted through other channels (Google Scholar), resulting in a total of 890 initial articles. After duplicate removing and screening according to the predefined criteria, a total of 12 eligible articles were selected (10, 13–23). Figure 1 summarizes the detailed information of article selection process. The study by Seong et al. (24) presented early physical therapy experience in patients with persistent pulmonary hypertension who underwent ECMO, but this study was a single case report and was therefore excluded. The study by Wells et al.'s study (25) also explored the safety and feasibility of early application of physical therapy in ECMO, but this study was a single cohort study without a control group and was therefore excluded. The study by Ko et al.'s study (26) did not compare the outcome measures of early RTI and delayed RTI and therefore was excluded. Eggmann et al. (27) compared the effects of early RTI and delayed RTI, but the rehabilitation training interventions were applied in the ICU, not specifically in patients undergoing ECMO, and the study was therefore excluded. We did not list all the excluded literature.

**Figure 1:**
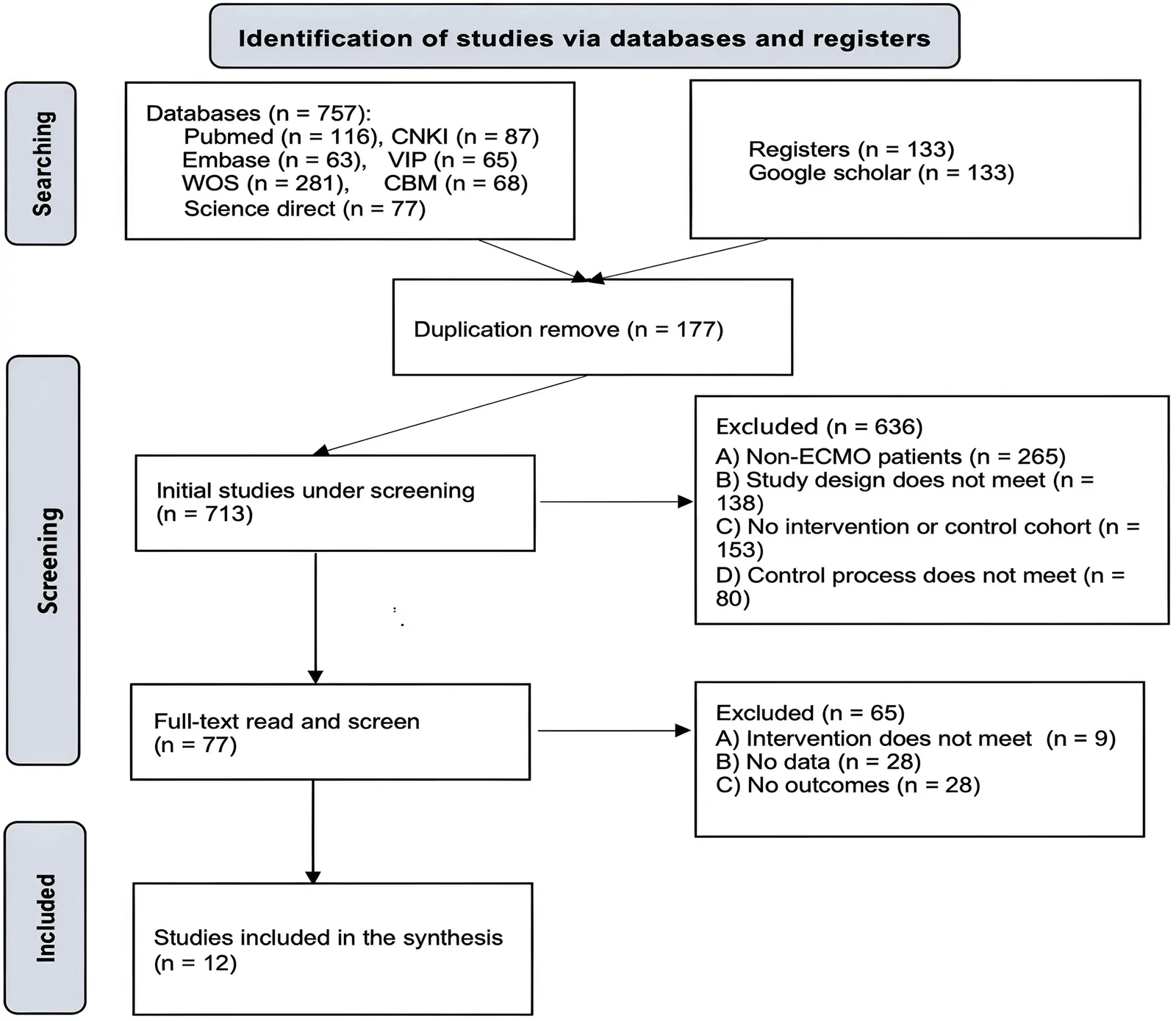
Flow diagram of the study selection process. A total of 890 records were initially identified (757 from online databases and 133 from other sources). After removing duplicates and screening according to predefined criteria, 12 eligible studies were included in the meta-analysis.

### Characteristics of the included studies

A total of 12 studies were included in our analysis, comprising five randomized controlled trials (10, 14, 16, 18, 23), four non-randomized controlled trials (13, 15, 21, 22), and three observational cohort studies (17, 19, 20). These studies were published between 2019 and 2024. The enrolled patients were primarily diagnosed with severe cardiac or respiratory conditions, including acute respiratory distress syndrome (ARDS), cardiac failure, fulminant myocarditis, COVID-19, and sepsis. The mean age of participants across the studies ranged from approximately 33 to 72 years.

The interventions in the included studies consisted of various early rehabilitation protocols, typically progressive and multidisciplinary. Most RTI included passive and active limb training, breathing exercises, resistance training, activities of daily living (ADL) training, and mobilization [including standing and walking exercises, as reported in one study (19)]. The definition of early RTI varied across the included studies; therefore, a broad definition based on the original study protocols was adopted. Rehabilitation interventions were initiated at different time points relative to ECMO support; specifically, initiation ranged from within 24 h of ECMO initiation (14) to (1–3) days after ECMO initiation (13), while some protocols commenced rehabilitation after ECMO decannulation (13, 14). Two studies defined early intervention as rehabilitation initiated within 7 days of ECMO support (19, 20). More details are described in Table 1.

**Table 1:** Basic characteristics, participants, interventions, and outcomes of included studies.

Studies	Publication year	Design	Diagnosis	Age (range)	ECMO type	E/C	RTI	Timing of RTI	Outcomes
Hayes et al. (10)	2020	RCT	ARDS, cardiac failure	52.0 ± 13.3	NR	7/8	A series of physical movements aimed at the highest level of activity that patients can tolerate	NR	Physiological parameters, mortality rate, total ECMO time, mechanical ventilation time, ICU time, first standing (walking) time
Yang et al. (13)	2023	NRCT	Severe heart disease patients	53.60 ± 15.73	NR	30/42	Progressive rehabilitation training program, from passive to active, activity intensity from weak to strong, and time from short to long	1–3 days after ECMO treatment in the intervention group, and 1–3 days after ECMO withdrawal in the control group	Serious complications during hospitalization, length of hospital stay, proportion of patients leaving the bed before discharge, and improvement rate
Zhang et al. (14)	2023	RCT	NR	44.22 ± 6.67	NR	20/18	Establish an early rehabilitation team, develop a plan, evaluate patients’ early rehabilitation activities, including passive training, active training, resistance training, and bedside activities	The intervention group received ECMO treatment within 24 h, while the control group received ECMO withdrawal	Physiological parameters, muscle strength score, incidence of adverse events
Xu et al. (15)	2021	NRCT	NR	58.64 ± 3.28	NR	41/41	Passive limb training, breathing training, resistance training	NR	Mechanical ventilation time, ICU time, hospitalization time, daily living ability
Ma et al. (16)	2023	RCT	Fulminant myocarditis	33. 49 ± 12. 64	NR	44/44	Passive activity during acute phase, active activity during recovery phase	NR	Physiological parameters, inflammatory cytokine levels, total ECMO time, mortality rate, cure rate
Mayer et al. (17)	2022	Retrospective cohort study	ARDS, cardiac failure	50 ± 14.6	VV-ECMO and VA-ECMO	218/70	NR	NR	ECMO total time, mechanical ventilation time, ICU time, hospitalization time, mortality rate, and 30-day readmission rate
George et al. (18)	2022	RCT	COVID-19	46.5 ± 8.5	VV-ECMO and VA-ECMO	23/19	﻿ NR	NR	ECMO total time and mortality rate
Cerier et al. (19)	2022	Retrospective single cohort study	ARDS	46.2 ± 13.7	VV-ECMO	30/37	Perform vertical exercise, suspension exercise, standing exercise, walking exercise, daily living activities, dynamic balance exercise, high-intensity aerobic exercise	The intervention group received ECMO treatment before 7 days, while the control group received ECMO treatment after 7 days	Physiological parameters, incidence of serious complications
Tonna et al. (20)	2023	Retrospective cohort study	COVID-19, congestive heart failure, chronic pulmonary disease	48.1 ± 13.7	VV-ECMO	1,830/6,330	NR	The intervention group received ECMO treatment before 7 days, while the control group received ECMO treatment after 7 days	Physiological parameters, total ECMO time, mortality rate
Liu et al. (21)	2019	NRCT	Sepsis, respiratory failure	72 (62–79)	NR	187/204	Physical therapy guided by multidisciplinary approach	NR	Length of hospital stay, mechanical ventilation time, daily living ability, mortality rate, ICU time, incidence of complications
Liu et al. (22)	2024	NRCT	NR	58.8 ± 14.27	VV-ECMO	23/22	A rehabilitation plan developed based on the patient's activity ability, which includes lung rehabilitation, functional exercise, nutritional support, and psychological therapy	NR	ECMO total time, mechanical ventilation time, ICU total time, mortality rate (survival rate)
Li et al. (23)	2021	RCT	Fulminant myocarditis	38.7 ± 11.6	NR	22/22	Corresponding physical therapy measures are taken for different stages of ECMO treatment	The intervention group received ECMO treatment, while the control group received ECMO withdrawal	Hospitalization time, cardiac function, maximum walking distance in 6 min

NRCT, non-randomized controlled study; ECMO, extracorporeal membrane oxygenation; VA-ECMO, venous-arterial extracorporeal membrane oxygenation; VV-ECMO, venous–venous extracorporeal membrane oxygenation; ARDS, acute respiratory distress syndrome; RTI, rehabilitation training intervention.

A wide range of outcomes was evaluated. Primary outcomes of interest included mortality rate (reported in eight studies (10, 13, 17–22), total duration of ECMO support [six studies (10, 16–19, 22)], and duration of mechanical ventilation [five studies (10, 15, 17, 21, 22)]. Secondary outcomes frequently assessed were length of stay in the ICU [five studies (10, 15, 17, 21, 22)] and total hospital length of stay [four studies (13, 15, 17, 21)]. Functional outcomes, such as muscle strength scores (14), activities of daily living (ADL) scores (15, 21), and the 6-min walk test distance (23), were also measured in some studies. Several studies also reported physiological parameters (10, 14, 16, 19, 20), incidence of complications (13, 19, 21), and other patient-centered metrics such as time to first ambulation (10). More details are provided in Table 1.

### Quality and bias assessment

In this study, some included studies (17–19) were observational cohort studies that lacked control groups. Among these studies, Cerier et al. (19) was a single-arm cohort study and was assessed as having a high risk of bias in the randomization process domain (per ROB 2.0 criteria, the observational designs carry such risks). Other studies (13, 15, 21, 22) were randomized controlled trials and did not describe the randomization process, resulting in a judgment of some concerns in the randomization domain. In addition, the studies (17–19) also showed some concerns regarding deviations from the intended interventions.

All included studies provided detailed documentation of participant dropouts, and no significant selective reporting bias or other biases were detected. Overall, six studies were rated as low-risk of bias, three as medium-risk studies, and three as high-risk studies. The overall methodological quality of the included studies was moderate. As shown in Figure 2A (risk of bias heat map across studies) and Figure 2B (overall bias distribution histogram), based on the Cochrane ROB 2.0 tool.

**Figure 2:**
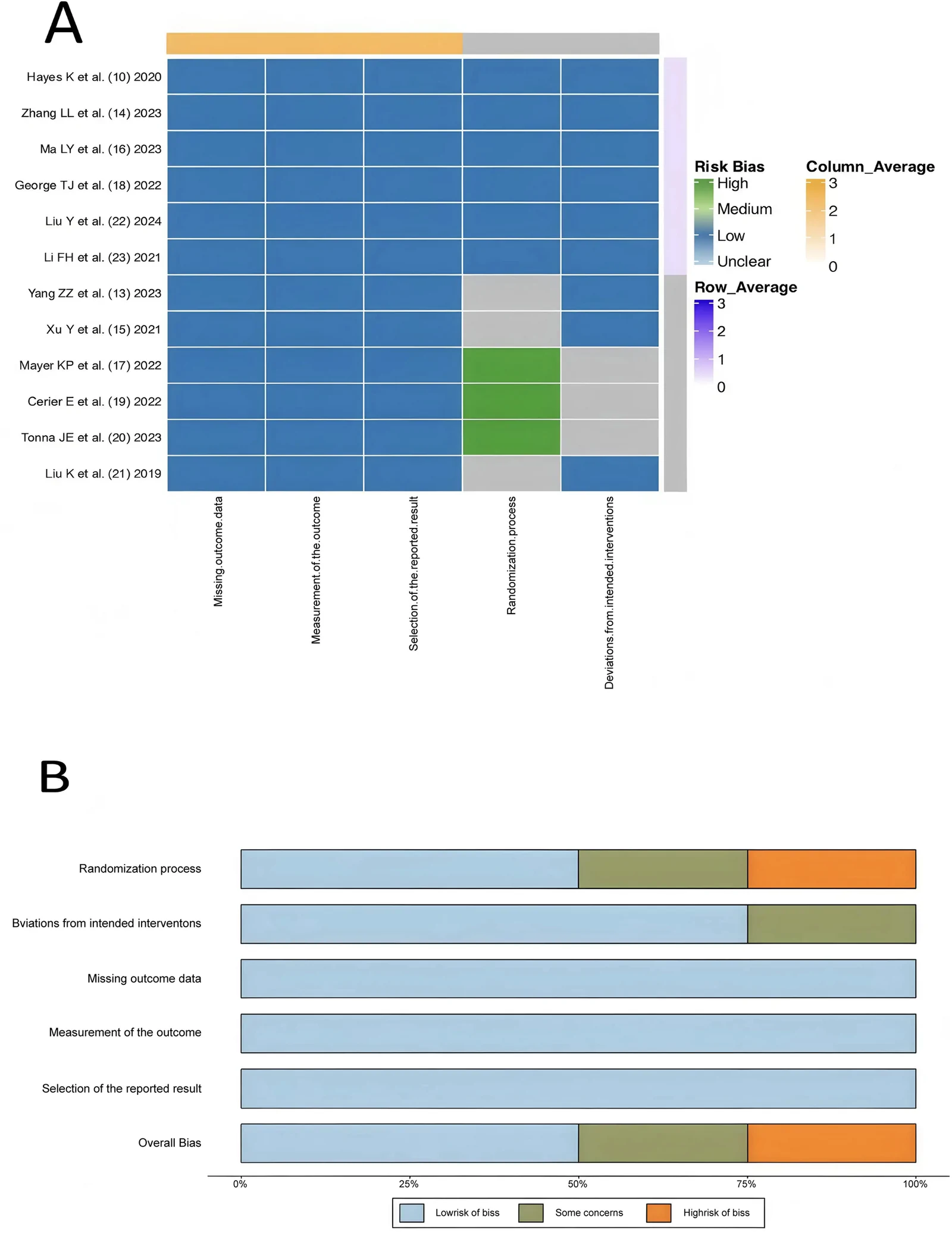
Risk of bias assessment of the included studies using the Cochrane ROB 2.0 tool. **(A)** Heat map showing the risk of bias for each included study across individual domains. **(B)** Summary histogram showing the overall distribution of risk of bias across all included studies. Blue, olive, and orange indicate low risk of bias, some concerns, and high risk of bias, respectively.

### Evaluation of rehabilitation outcomes

#### Hospital length of stay

Seven of the 12 articles reported data on hospital LOS comparing early and delayed RTI, and significant statistical heterogeneity was observed (*I*^2^ = 92%, Cochrane *P* < 0.01). Using a random effects model, the pooled meta-analysis found no significant difference in hospital LOS between the early and delayed RTI groups [MD = −2.69, 95% CI (−7.22; 1.84), *Z* = −1.17, *P* = 0.24] (Figure 3A).

**Figure 3:**
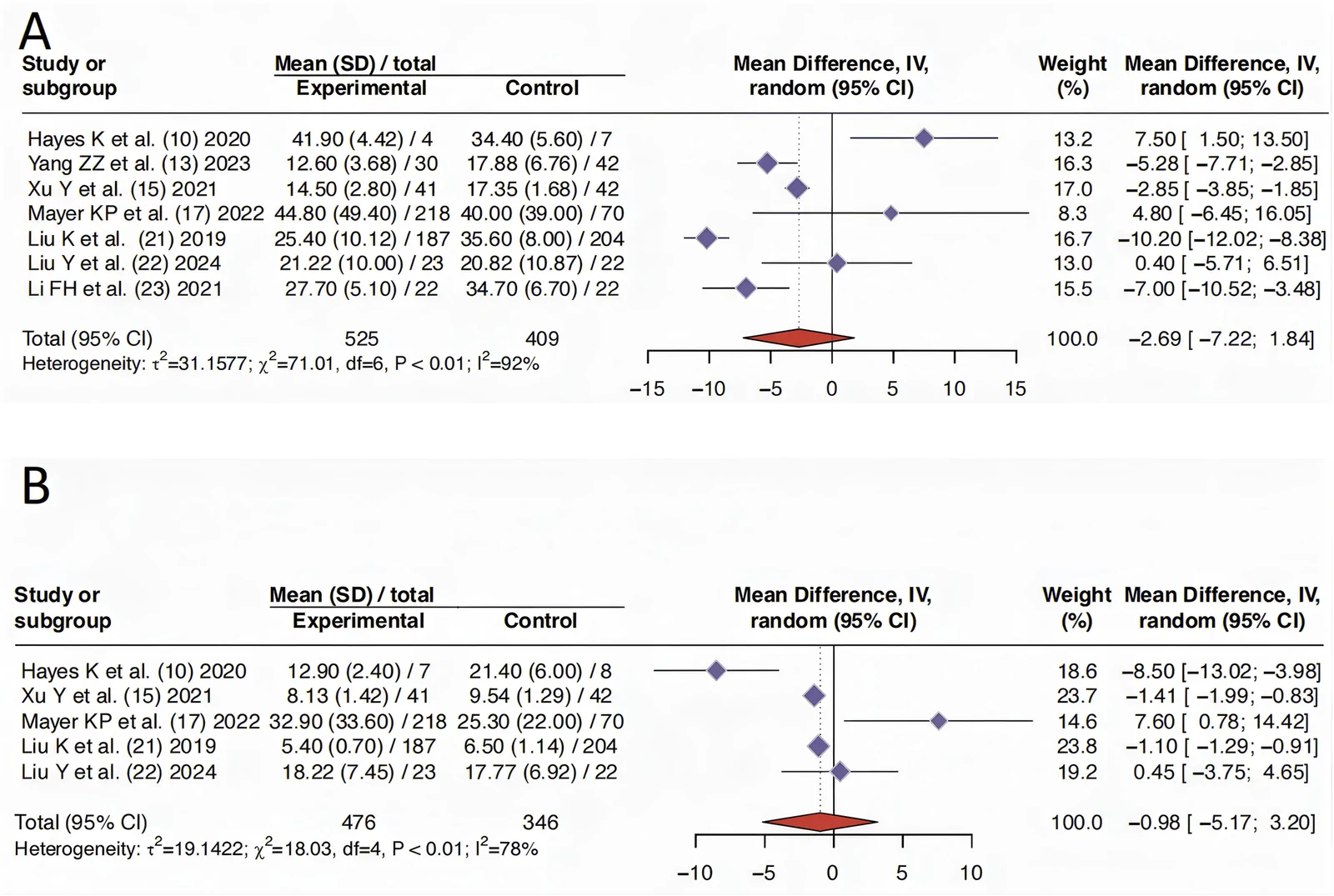
Forest plots of length of stay outcomes comparing early versus delayed rehabilitation therapy (RTI) in patients receiving ECMO. **(A)** Hospital length of stay (LOS). **(B)** Intensive care unit (ICU) LOS. Pooled effect sizes were calculated using a random-effects model. Squares represent the effect size of each study, with the size proportional to the study weight; horizontal lines indicate 95% confidence intervals; diamonds represent the overall pooled estimates. ECMO, extracorporeal membrane oxygenation; RTI, rehabilitation therapy; LOS, length of stay; ICU, intensive care unit; CI, confidence interval.

#### ICU length of stay

Five articles reported ICU LOS and significant heterogeneity was also present (*I*^2^ = 78%, Cochrane *P* < 0.01). The random-effects meta-analysis indicated no significant difference in ICU LOS between groups [MD = −0.98, 95% CI (−5.17; 3.20), *Z* = −0.46, *P* = 0.64] (Figure 3B).

#### Duration of mechanical ventilation and ECMO support

Five studies compared the duration of mechanical ventilation between early and delayed RTI. Significant statistical heterogeneity was detected (*I*^2^ = 84%, Cochrane *P* < 0.01). A random-effects meta-analysis model showed no significant difference in mechanical ventilation duration between early and delayed RTI [MD = −0.50, 95% CI (−2.62; 1.63), *Z* = −0.46, *P* = 0.65] (Figure 4A).

**Figure 4:**
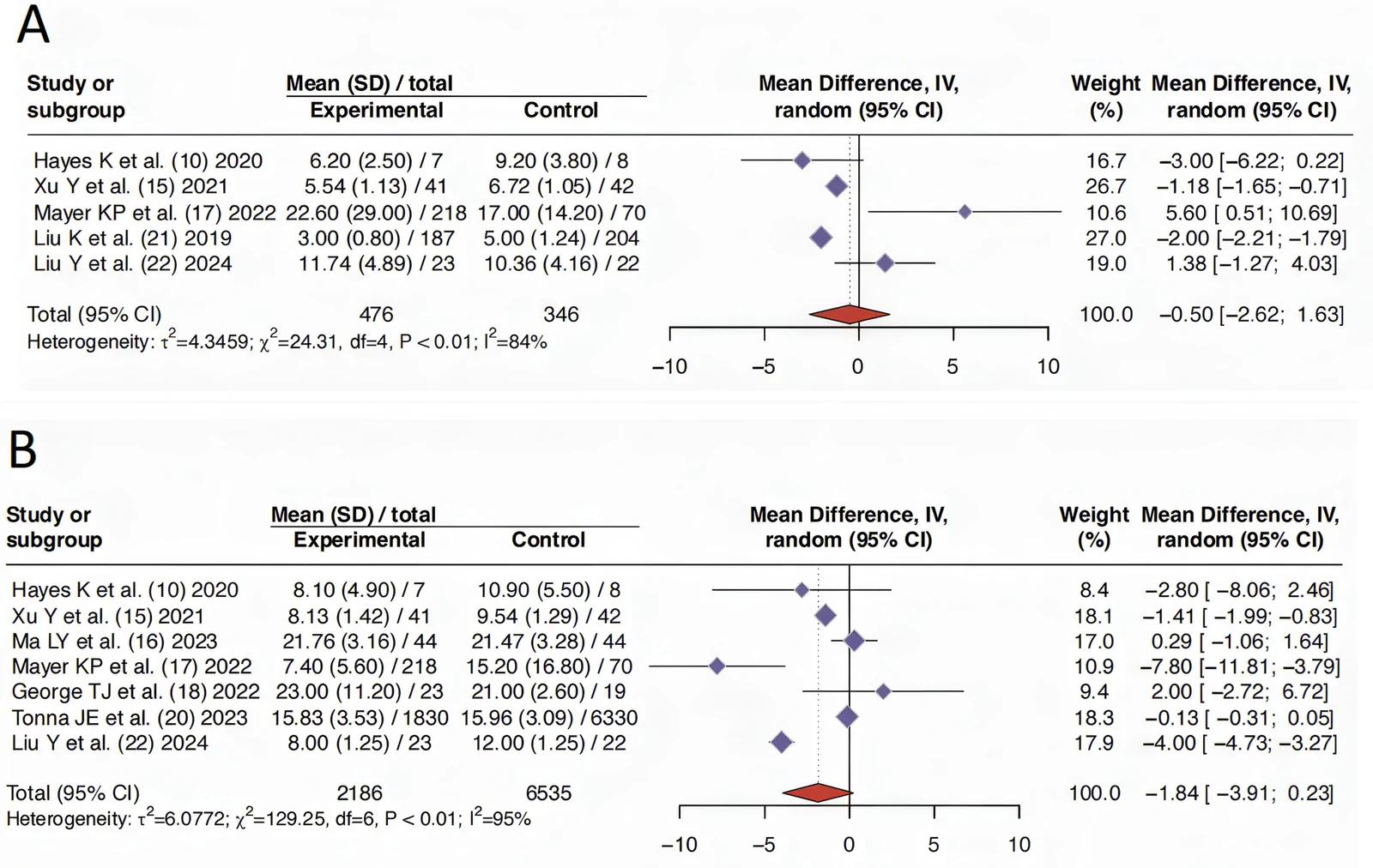
Forest plots of ventilation and ECMO support durations comparing early versus delayed rehabilitation therapy (RTI). **(A)** Duration of mechanical ventilation. **(B)** Duration of ECMO support. Pooled effect sizes were calculated using a random-effects model. Squares represent the effect size of each study, with the size proportional to the study weight; horizontal lines indicate 95% confidence intervals; diamonds represent the overall pooled estimates. ECMO, extracorporeal membrane oxygenation; RTI, rehabilitation therapy; CI, confidence interval.

Seven studies reported on ECMO duration between early and delayed RTI. Considerable heterogeneity was observed (*I*^2^ = 95%, Cochrane *P* < 0.01). Using a random-effects model, the pooled meta-analysis indicated that the difference in ECMO duration between early and delayed RTI groups was not statistically significant [MD = −1.84, 95% CI (−3.91; 0.23), *Z* = −1.74, *P* = 0.08] (Figure 4B).

#### In-hospital mortality and adverse event rate

A total of seven studies reported on in-hospital mortality between early and delayed RTI, with statistical heterogeneity (*I*^2^ = 73%, Cochrane *P* < 0.01). Using a random-effects model, the meta-analysis showed no significant difference in mechanical ventilation time between early and delayed RTI [RR = 0.76, 95% CI (0.51; 1.12), *Z* = −1.41, *P* = 0.16] (Figure 5A).

**Figure 5:**
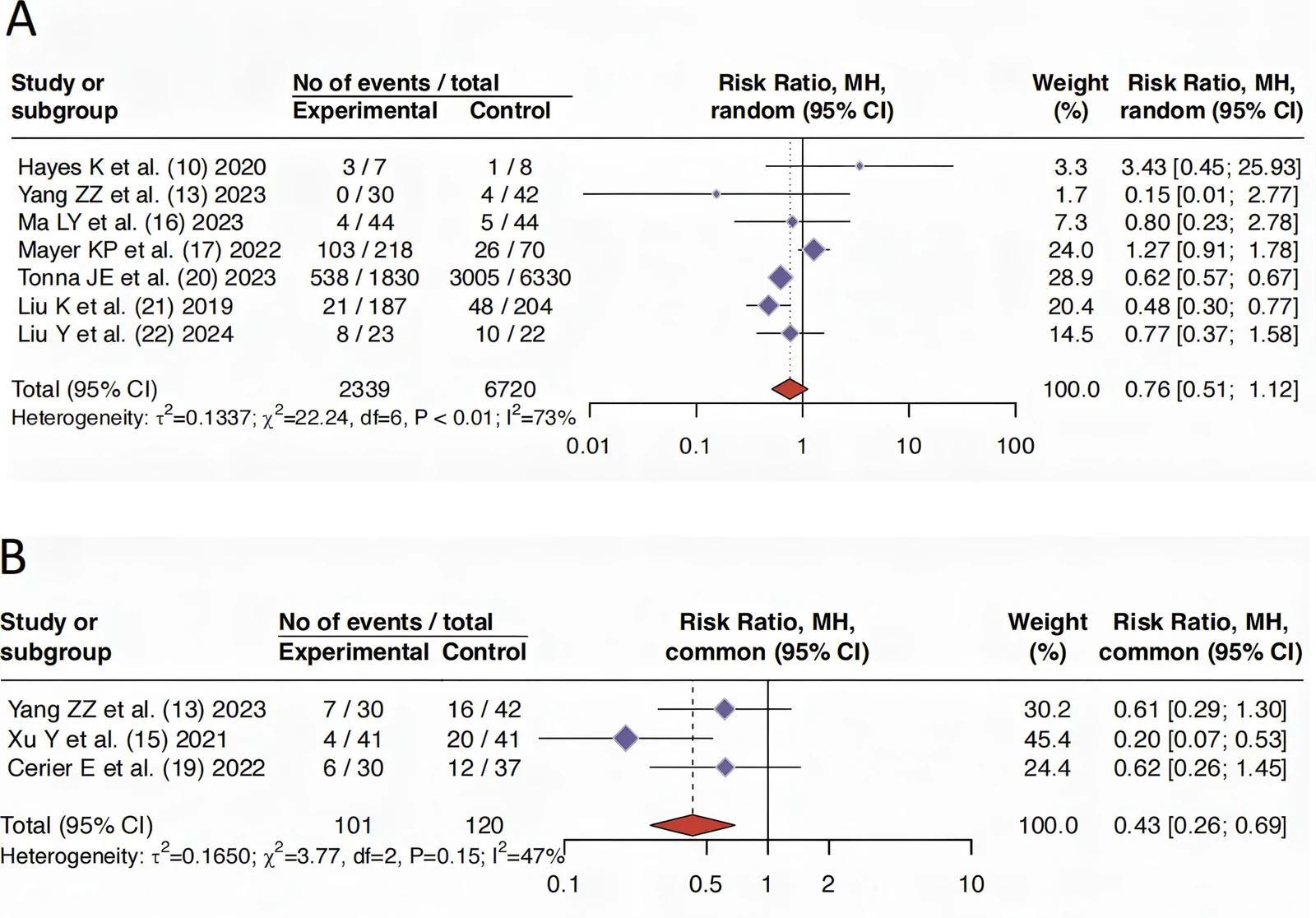
Forest plots of mortality and adverse event outcomes comparing early versus delayed rehabilitation therapy (RTI). **(A)** In-hospital mortality. **(B)** Adverse event rate. Pooled effect sizes were calculated using a random-effects model. Squares represent the effect size of each study, with the size proportional to the study weight; horizontal lines indicate 95% confidence intervals; diamonds represent the overall pooled estimates. RTI, rehabilitation therapy; CI, confidence interval.

Three articles reported the adverse event rates of early and delayed RTI, with statistical heterogeneity among the studies (*I*^2^ = 95%, Cochrane *P* < 0.01). Using a random-effects model, the meta-analysis showed no significant difference in the adverse event rates between early and delayed physical therapy [RR = 0.43, 95% CI (0.26; 0.69), *Z* = −3.48, *P* < 0.001] (Figure 5B).

#### Sensitivity analysis

In the meta-analysis of hospital LOS, the removal of the study by Hayes et al. (10), resulted in a statistically significant pooled result, suggesting that this study had a significant impact on the overall outcome (Figures 6A, B). This effect is likely attributable to its relatively small size. After excluding this study, pooled effect sizes of hospital LOS, ICU LOS, mechanical ventilation duration, ECMO duration, and In-hospital mortality were recalculated (Table 2).

**Figure 6:**
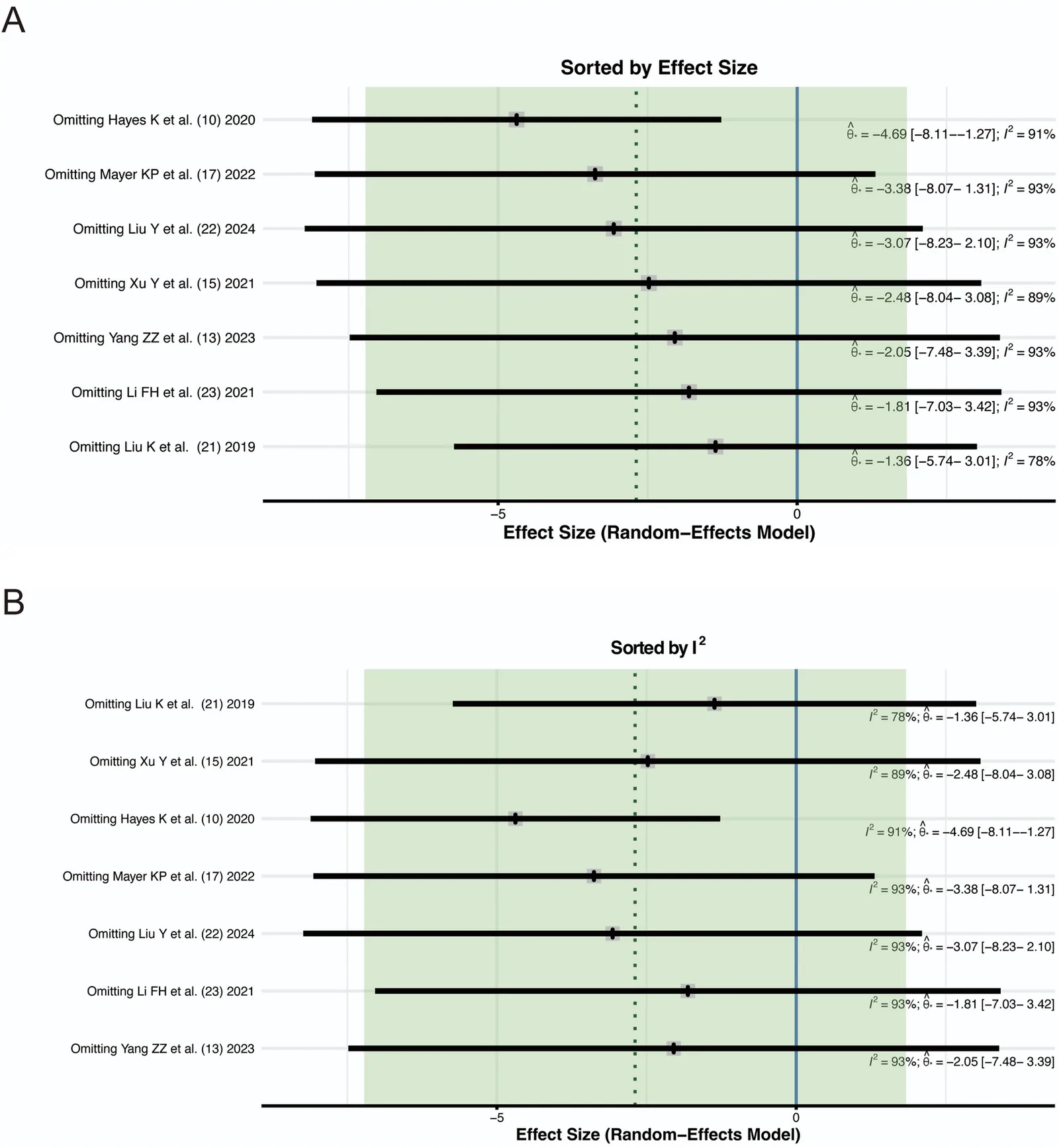
Sensitivity analysis for hospital length of stay and other outcomes after excluding one study. **(A)** Forest plot of hospital length of stay before and after exclusion of the study. **(B)** Recalculated pooled effect estimates for ICU length of stay, mechanical ventilation duration, ECMO duration, and in-hospital mortality after exclusion. All analyses were performed using a random-effects model. Squares represent effect sizes; horizontal lines indicate 95% confidence intervals; diamonds represent pooled estimates. ECMO, extracorporeal membrane oxygenation; ICU, intensive care unit; CI, confidence interval.

**Table 2:** Adjusted pooled effect size of outcome indicators.

Index	Outcome	Number of studies included	Effect size	Heterogeneity		*Z*, *P*-value
				*I* ^2^	*P*	
1	Hospital LOS	6	MD = −4.69, 95% CI [−8.11; −1.27]	91%	<0.01	*Z* = −2.69, *P* = 0.007
2	ICU LOS	4	MD = −1.12, 95% CI [−1.30; −0.94]	61.6%	0.05	*Z* = −12.41, *P* < 0.001
3	Mechanical ventilation time	4	MD = 0.14, 95% CI [−2.48; 2.77]	87.4%	<0.01	*Z* = 0.11, *P* = 0.914
4	ECMO duration	6	MD = −1.77, 95% CI [−4.10; 0.56]	96.1%	<0.01	*Z* = −1.49, *P* = 0.136
5	In-hospital mortality	6	RR = 0.72, 95% CI [0.49; 1.06]	74.5%	<0.01	*Z* = −1.66, *P* = 0.097
6	Adverse event rate	3	RR = 0.43, 95% CI [0.26; 0.69]	95%	<0.01	*Z* = −3.48, *P* < 0.001

#### Publication bias

To assess potential publication bias, funnel plots were constructed for the outcomes of hospital LOS and ICU LOS (Figures 7A, B). Egger's regression test results (*P* = 0.78 and 0.90, respectively) indicated that there was no asymmetry on either side of the funnel plots.

**Figure 7:**
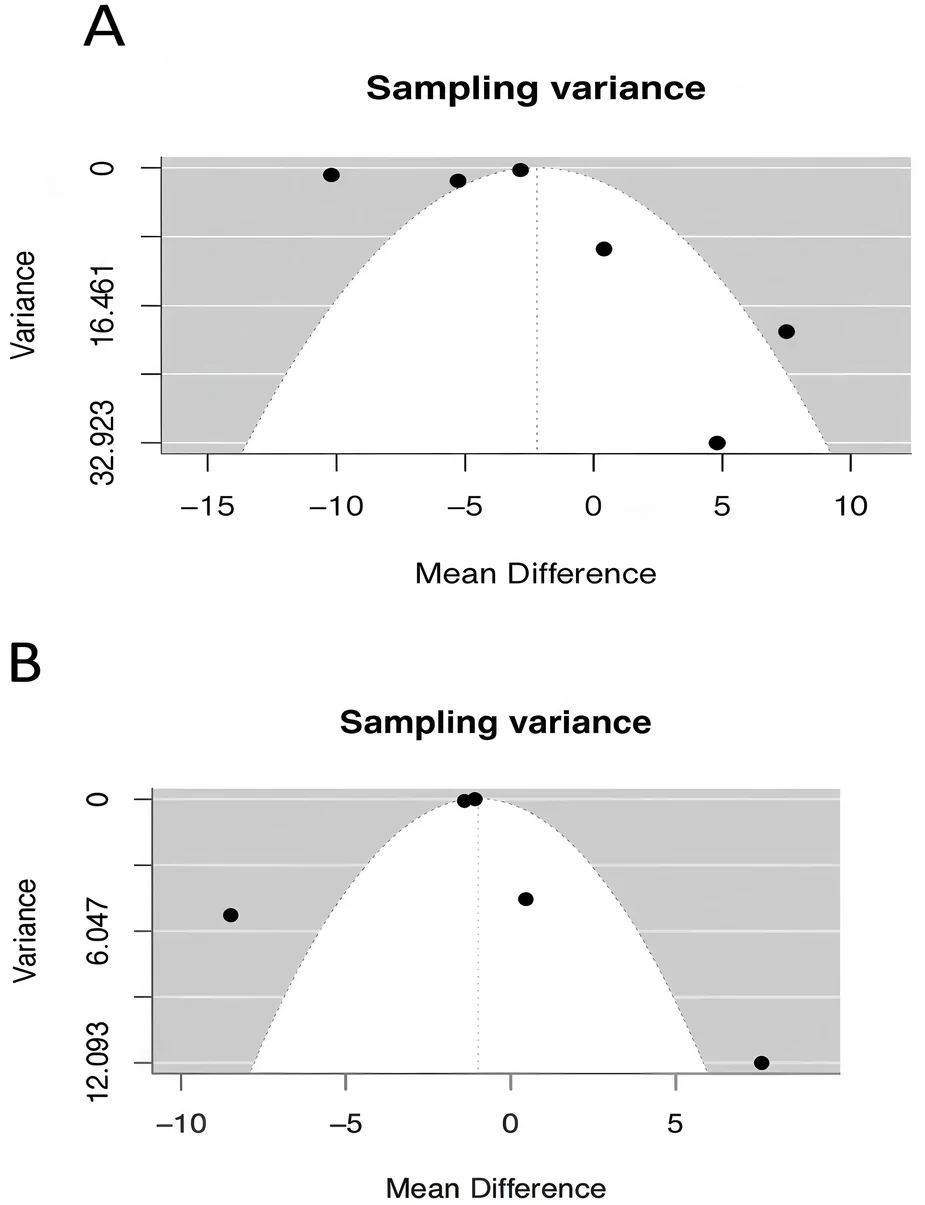
Funnel plots for assessing publication bias. **(A)** Hospital length of stay. **(B)** ICU length of stay. Egger's regression test showed no significant asymmetry (*P* = 0.78 for hospital LOS and *P* = 0.90 for ICU LOS, respectively). LOS, length of stay; ICU, intensive care unit.

## Discussion

This systematic review and meta-analysis of 12 studies evaluated the effects of early RTI in patients undergoing ECMO. The pooled results indicated that early RTI did not significantly reduce hospital LOS, ICU LOS, duration of mechanical ventilation, ECMO support, or in-hospital mortality compared with delayed rehabilitation. However, sensitivity analysis after excluding one small-sample RCT (19) revealed statistically significant reductions in both hospital and ICU LOS in the early RTI group. Moreover, early RTI was associated with a significantly lower incidence of adverse events. These findings suggest that while early rehabilitation may offer benefits in reducing complications and potentially shortening length of stay under certain conditions, its effects on other critical outcomes remain uncertain given the current evidence.

Prolonged bed rest and the use of high-dose sedatives and muscle relaxants are known to increase the risk of disability in ECMO survivors (28). Although early RTI has the potential to enhance cardiac function, improve exercise endurance, reduce overall hospitalization time, and improve patient survival, its effectiveness still lacks systematic evaluation. Significant heterogeneity was observed across the 12 studies regarding ICU LOS and hospital LOS. These heterogeneities can be contributed to factors such as study design, patient characteristics, control processes, measurement of outcome data, etc. Notably, the study by Cerier et al. (19) focused exclusively on COVID-19 patients and reported that early RTI significantly improved activity measure scores but did not significantly shorten the overall hospital stay.

The timing of early RTI initiation varied considerably across the 12 studies. Studies (13, 14) reported that early RTI in the intervention group began 1–3 days after ECMO initiation, while physical therapy in the control group began after ECMO withdrawal. Conversely, studies (19, 20) reported that early RTI in the intervention group was initiated within 7 days after ECMO initiation, whereas physical therapy in the control group began 7 days post-ECMO. One study (23) provided early RTI during ECMO support in the intervention group, while the control group started after ECMO withdrawal. Currently, there are no universally accepted guidelines regarding the optimal timing for early RTI. The Extracorporeal Life Support Organization (29) recently (2024) suggested that early physical therapy may be initiated either before or after intubation once the patient is hemodynamically stable and adequately sedated. We recommend that clinicians individualize RTI timing based on the patient's consciousness level, oxygen demand (PaO_2_/FiO_2_), and dependence on respiratory support.

The outcomes reported by the 12 studies on the application of early physical therapy in ECMO also varied. Furthermore, hospital LOS was only reported in seven studies (10, 13, 15, 17, 21–23) while not available in other studies. ICU LOS was reported in five studies (10, 15, 17, 21, 22), but not in the remaining studies. The pooled results indicated no significant difference in both hospital LOS and ICU LOS between early and delayed RTI. However, in the sensitivity analysis, excluding study (10) revealed a statistical significant difference between the two groups. It is speculated that the results may be influenced by the inclusion of the pilot RCT literature with a small sample size. However, after excluding the literature (10), an adjusted analysis of the remaining literature was conducted. The results after excluding this study showed that the hospital LOS and ICU LOS of the intervention group were significantly shorter than those of the control group.

We propose several mechanisms through which early RTI may confer benefits. It may reduce the incidence of ventilator-associated pneumonia, improve static lung compliance, enhance secretion clearance, prevent atelectasis, and mitigate muscle atrophy and weakness related to immobility and sedation (30). In addition, prolonged deep sedation and the use of nerve blockers often lead to significant muscle atrophy and weakness in patients receiving ECMO, whereas early initiation of RTI can help mitigate neuromuscular weakness and reduce complications associated with extended bed rest (27). Notably, functional improvements were also evident: one study (23) compared the maximum walking distance within 6 min after ECMO between early and delayed RTI and found that patients in the early RTI demonstrated better physical function. Similarly, one study (14) assessed the muscle strength of the two groups and found that on day 7 after treatment, the muscle strength score of the early RTI group was significantly higher than that of the control group. ECMO therapy increases the risk of developing pressure injuries, ventilator-associated pneumonia, acquired muscle weakness, deep vein thrombosis, and other serious complications (31). Among the 12 included studies, three (13, 15, 19) reported complication rates, and the meta-analysis indicated a significantly lower incidence in the early RTI groups, supporting the role of early physical therapy in reducing these adverse events.

However, no significant difference was detected between the RTI group and the control group in duration of mechanical ventilation, ECMO duration, or in-hospital mortality. This suggests that while early RTI offers functional and clinical benefits, it has limited effect on the duration of recovery. In clinical practice, safety is another critical consideration. Early RTI is more commonly used in patients who are awake and have not undergone endotracheal intubation. Patients with spontaneous breathing are better candidates to engage in early activities such as sitting and walking (32). During functional exercise, patients receiving ECMO face risks such as bleeding at the cannulation site, disconnection or loosening at circuit connections, or hemodynamic shifts from frequent position changes, which may lead to a decrease in blood flow to the membrane oxygenator, endangering the patient's life (33). Although adverse events during functional exercise are rare, there are still potential risks (25).

Early RTI appears applicable across ECMO modalities. In this meta-analysis, studies (19, 20, 22) included only patients receiving VV-ECMO, while studies (17, 18) included both patients receiving VV-ECMO and VA-ECMO. The ECMO cannula inserted through the femoral artery has a large diameter and limits hip flexibility, increasing the risk of complications such as bleeding at the puncture site and cannula displacement, which may pose a contraindication for active mobilization (34). Moreover, the placement of a VV-ECMO inguinal canal can also limit the patient's ability to engage in rehabilitation training (35). Therefore, for both VV-ECMO and VA-ECMO, ensuring safety during functional exercise is a primary consideration during the implementation of early rehabilitation. Studies (10, 16, 19, 20) documented that during RTI, physiological parameters such as blood pressure, heart rate, and SpO_2_ should be closely monitored to ensure safety.

Early RTI can be adapted to individual patient needs. Staged rehabilitation protocols, such as those described by Li et al. (23), which implemented early RTI during ECMO support, weaning, and post-decannulation phases, demonstrate that tailored interventions are feasible across the treatment continuum. Although active exercise, respiratory muscle training, psychological support, and other rehabilitation measures may not be feasible during the early deep sedation stage of ECMO treatment, clinical nursing staff can still promote patient recovery through strategies such as posture management, enhanced sputum drainage, neuromuscular electrical stimulation, and nutritional support. Supine position muscle strength repair and strengthening exercises, such as active/passive joint range of motion training and resistance training, can also provide benefits for maintaining muscle strength. Although the Extracorporeal Life Support Organization (29) and other guidelines have not provided a specific definition for early RTI protocols, they emphasize that early RTI should be progressively tailored to patients’ capabilities, involving a transition from passive exercise to more active interventions such as sitting up and walking.

### Limitations

This study represents the first systematic review and meta-analysis to evaluate the effect of rehabilitation interventions for patients supported by ECMO through meta-analysis helping to clarify the ongoing debate regarding when to initiate RTI. Several limitations should be acknowledged. The small number of included studies (*n* = 12) limited the robustness of the conclusions. Substantial statistical heterogeneity was observed across all pooled outcomes (*I*^2^ = 73%–95%), which may be attributable to differences in study design, patient populations (VV-ECMO vs. VA-ECMO and heterogeneous underlying diseases), RTI protocols (timing, modality, intensity, and duration), and outcome definitions. Although sensitivity analysis excluding a single small study rendered hospital LOS statistically significant, heterogeneity remained high, indicating that multiple factors contributed. Due to the limited number of studies per outcome (3–7), subgroup analyses stratified by RTI modality or initiation timing were not feasible, as most subgroups would contain only one to two studies. Significant heterogeneity in outcome reporting precluded meta-analysis of several patient-centered endpoints, such as activities of daily living (e.g., Barthel Index), functional exercise capacity (6-min walk distance), muscle strength, 30-day readmission rates, and long-term quality-of-life. Furthermore, the methodological quality of included studies was generally moderate to low; observational studies lacked control groups, and RCTs methodological deficiencies in randomization (e.g., inadequate description of allocation concealment) and allocation concealment. The included studies had marked clinical heterogeneity with differences in disease phenotypes, severity stratification, and rehabilitation protocols ; this limits the generalizability of the findings.

## Conclusions

Compared with delayed rehabilitation, early RTI in ECMO patients is associated with a significant reduction in complication rates and, in sensitivity analysis, a reduction in both ICU and hospital length of stay. However, it did not significantly affect the duration of mechanical ventilation, ECMO support, or in-hospital mortality. Given the overall moderate-to-low quality of the available evidence and high statistical heterogeneity, these findings should be interpreted cautiously. Well-designed, larger-scale RCTs are needed to establish optimal RTI timing and strategies and to confirm the benefits of early mobilization in this vulnerable population.

## Data Availability

The original contributions presented in the study are included in the article/Supplementary Material, further inquiries can be directed to the corresponding authors.
